# Retrospective Evaluation of Platelet-Leukocyte Indices and Cardiac Surgical Outcomes in Acyanotic Heart Disease Patients with Pulmonary Hypertension (REPLICA-PH)

**DOI:** 10.21470/1678-9741-2020-0648

**Published:** 2022

**Authors:** Ashish Walian, Jasvinder Kaur Kohli, Rohan Magoon, Ramesh Chand Kashav, ItiShri, Souvik Dey, Narender Singh Jhajhria

**Affiliations:** 1 Department of Cardiac Anaesthesia, Atal Bihari Vajpayee Institute of Medical Sciences (ABVIMS) and Dr. Ram Manohar Lohia Hospital, Baba Kharak Singh Marg, New Delhi, India; 2 Department of Cardiothoracic and Vascular Surgery, Atal Bihari Vajpayee Institute of Medical Sciences (ABVIMS) and Dr. Ram Manohar Lohia Hospital, Baba Kharak Singh Marg, New Delhi, India

**Keywords:** Heart Defects, Congenital, Neutrophil-Lymphocyte, Platelet-Lymphocyte, Postoperative Complications, Inflammation, Cardiac Output, Low, Blood Pressure

## Abstract

**Introduction:**

Acyanotic congenital heart disease (ACHD) patients with pulmonary hypertension (PH) are prone to postoperative complications, and characterization of the risk profile continues to fail in identifying inflammatory predilection. Our objective is to investigate the role of platelet-leukocyte indices (neutrophil-lymphocyte ratio [NLR], platelet-lymphocyte ratio [PLR], and systemic immune-inflammation index [SII] [neutrophil × platelet/lymphocyte]) in predicting poor outcomes following cardiac surgery in ACHD cohort with preoperative PH.

**Methods:**

This single-center, retrospective risk-predictive study included ACHD patients undergoing surgical correction at our tertiary cardiac center between January 2015 and December 2019. Standard institutional perioperative management protocol was followed, and poor postoperative outcome was defined as ≥ 1 of: low cardiac output syndrome, new-onset renal failure, prolonged mechanical ventilation (MV > 24 hours), stroke, sepsis, and/or death.

**Results:**

One hundred eighty patients out of 1,040 (17.3%) presented poor outcome. On univariate analysis, preoperative factors including right ventricular systolic pressure (RVSP) (PH-severity marker), congestive heart failure, albumin, NLR, PLR, SII, and aortic cross-clamping (ACC) and cardiopulmonary bypass (CPB) times predicted poor outcome. However, on multivariate analysis, RVSP, NLR, SII, and ACC and CPB times emerged as independent predictors. An NLR, SII prognostic cutoff of 3.33 and 860.6×103/mm3 was derived (sensitivity: 77.8%, 78.9%; specificity: 91.7%, 82.2%; area under the curve: 0.871, 0.833). NLR and SII values significantly correlated with postoperative MV duration, mean vasoactive-inotropic scores, and length of intensive care unit and hospital stay (*P*<0.001).

**Conclusion:**

Novel parsimonious, reproducible plateletleukocyte indices present the potential of stratifying the risk in congenital cardiac surgical patients with pre-existing PH.

**Table:** 

Abbreviations, acronyms & symbols
**ACC = Aortic cross-clamping** **ACHD = Acyanotic congenital heart disease** **ARF = Acute renal failure** **ASD = Atrial septal defect** **AUC = Area under the curve** **AVSD = Atrioventricular septal defect** **CCF = Congestive cardiac failure** **CI = Confidence interval** **COPD = Chronic obstructive pulmonary disease** **CPB = Cardiopulmonary bypass** **CRP = C-reactive protein** **DLC = Differential leukocyte count** **DO-MV = Duration of mechanical ventilation** **ECMO = Extracorporeal membrane oxygenation** **F = Favorable outcome** **Hb = Hemoglobin** **LOS-H = Length of hospital stay** **LOS-ICU = Length of intensive care unit stay** **MV = Mechanical ventilation** **NLR = Neutrophil-lymphocyte ratio** **OR = Odds ratio** **P = Poor outcome** **PH = Pulmonary hypertension** **PLR = Platelet-lymphocyte ratio** **ROC = Receiver operating characteristic** **RVSP = Right ventricular systolic pressure** **SII = Systemic immune-inflammation index** **TLC = Total leukocyte count** **VIS = Vasoactive-inotropic score** **VSD = Ventricular septal defect**

## INTRODUCTION

Our comprehension of pulmonary hypertension (PH) has evolved over the last decade to suggest an important role of inflammation in perpetuating the vasculopathy associated with a severe form of disease^[1]^. The liaison between the immune and inflammatory mechanisms in PH is heralded by the infiltration of inflammatory cells, growth factors, cytokines, and chemokines in the remodeled vasculature^[1]^. As an extension of the aforementioned, the surgical cohort of acyanotic congenital heart disease (ACHD) patients with preoperative PH (primarily owing to an increased pulmonary blood flow with an underlying left-right shunt) constitutes a peculiarly predisposed subset where in the preexisting PH-associated inflammation is compounded by the inexorable systemic inflammatory response to cardiopulmonary bypass (CPB)^[2]^. Considering the fact that most of the corrective congenital cardiac surgeries mandate the use of CPB, the subsequent postoperative outcome is intricately influenced by the cumulative inflammatory response syndrome^[3,4,5,6]^. While the ACHD patients with PH are inherently prone to pulmonary hypertensive crisis, difficult weaning from CPB, and major organ complications in the postoperative period, the characterization of the risk profile continues to fail in identifying inflammatory predilection^[7]^.

There is an increasing trend towards evaluating the prognostic role of novel, readily available hematological pro-inflammatory markers in cardiac patients given the involved cost limits the routine performance of established markers of inflammation, such as high sensitivity-complement reactive protein and procalcitonin. In this context, a considerable literature is accumulating on the utility of these parsimonious hematological indices (including neutrophil-lymphocyte ratio [NLR], platelet-lymphocyte ratio [PLR], and systemic immune-inflammation index [SII] [neutrophil × platelet/lymphocyte]) in predicting outcomes in adult cardiac operative^[8,9,10]^ and non-operative settings^[11,12]^. Nevertheless, few initial research reports have highlighted the prognostic role of NLR in well-defined congenital cardiac subset undergoing palliative procedures such as Norwood operation^[13]^ and bidirectional Glenn shunt^[14]^. Interestingly, these hematological indices were also recently described to be elevated in chronic obstructive pulmonary disease (COPD) patients complicated with PH while suffering an acute exacerbation episode, motivating the pediatric cardiac anesthesia-surgical team at our tertiary care center to examine a possible association between these novel inflammatory indices and outcomes in operative settings of patients ailing from preoperative PH^[15]^. Therefore, the present study was contemplated to stage a “Retrospective Evaluation of Platelet-Leukocyte Indices (in context of predictive performance) and Cardiac surgical outcomes in Acyanotic heart disease patients with Pulmonary Hypertension” (or REPLICA-PH).

## METHODS

Following a formal approval from the institutional ethics committee board (No.388 (37/2020) IEC/ABVIMS/RMLH), the study was conducted at our tertiary care referral center. The hospital record archive files and/or electronic database were retrospectively analyzed.

**Fig. 1:**
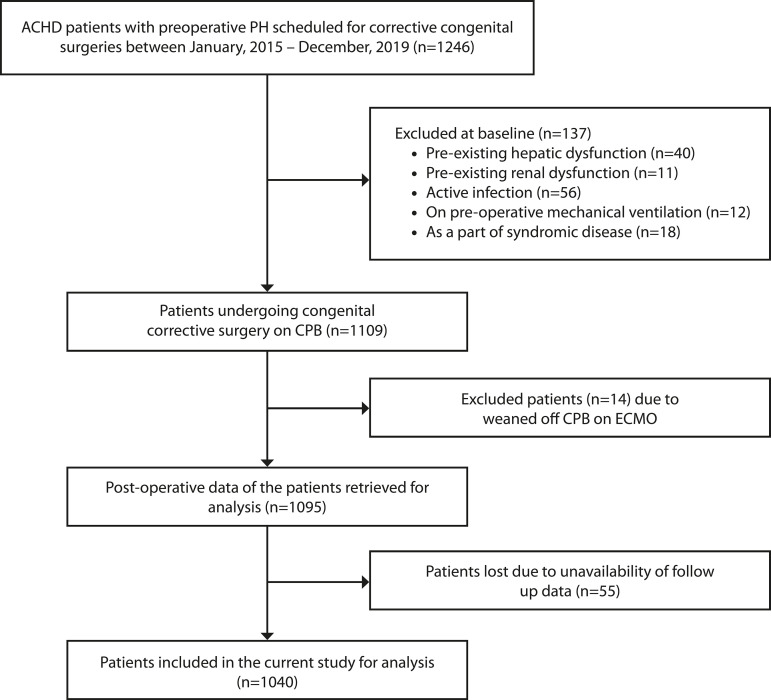
Flow chart diagram for patient enrolment. ACHD=acyanotic congenital heart disease; CPB=cardiopulmonary bypass; ECMO=extracorporeal membrane oxygenation; PH=pulmonary hypertension

A total of 1,246 consecutive ACHD patients (under 18 years of age and with a diagnostic association of preoperative PH) undergoing elective corrective cardiac surgery on extracorporeal CPB support between 1^st^ of January 2015 and 31^st^ of December 2019 were primarily included in the index study. The patients with any of the following conditions were subsequently excluded: pre-existing anaemia (hemoglobin [Hb] < 9 g/dl), requiring mechanical ventilation preoperatively, inability to retrieve total leukocyte count (TLC) and differential leukocyte count (DLC) data, presence of active infection in the immediate preoperative period, concomitant corticosteroid therapy, known immunosuppressive disease, multisystem syndromic association, preoperative hepatic and renal dysfunction, and patients post-correction weaned from CPB on extracorporeal membrane oxygenation. Thereafter, another 55 patients were excluded from the study due to unavailability of follow-up data. Finally, 1,040 patients were evaluated for postoperative outcome. The flow diagram for patient enrolment is illustrated in Figure 1.

Preoperative clinical and demographic characteristics of the patients such as age, sex, weight, height, documented right ventricular systolic pressure (RVSP) (as an estimate of pulmonary artery systolic pressure, thereby classifying the PH severity), primary diagnosis of ACHD, and history of preoperative congestive cardiac failure (CCF) were noted. The underlying preoperative PH in the subjects was stratified as per the joint task force nosology put forth by the European Society of Cardiology (or ESC) and the European Respiratory Society (or ERS) for PH management^[16]^:

(i) Mild PH: RVSP ranging between 36 and 50 mmHg;
(ii) Moderate PH: RVSP ranging between 51 and 70 mmHg;
(iii) Severe PH: RVSP > 70 mmHg.

The laboratory data obtained for every participant within 48 hours preoperatively was considered for the subsequent analysis and includes Hb, TLC, DLC, platelet count, blood urea, serum creatinine, and albumin. Using the available information in the DLC, NLR (neutrophil count/lymphocyte count), PLR (platelet count/lymphocyte count), and SII (platelet count × neutrophil count/lymphocyte count), values were computed. The intraoperative aortic cross-clamping (ACC) time and CPB time were also registered for all the study participants.

The following postoperative parameters were also evaluated in the present study: vasoactive-inotropic score (VIS) (dopamine [µg/kg/min] + dobutamine [µg/kg/min] + milrinone [µg/kg/min] × 10 + epinephrine [µg/kg/min] × 100 + norepinephrine [µg/kg/ min] × 100 + vasopressin [units/kg/min] × 10000)^[10]^, postoperative duration of mechanical ventilation (DO-MV), length of intensive care unit stay (LOS-ICU), and length of hospital stay (LOS-H). The development of poor postoperative outcome was defined by ≥ 1 of the following: low cardiac-output syndrome (cardiac index < 1.5 L/min/m^2^), acute renal failure (ARF), postoperative DO-MV (> 24 hours), stroke, sepsis, and death within 30 days postoperatively; these constituted the primary endpoint of the study. Postoperative ARF was diagnosed in accordance with the pediatric modified Risk, Injury, Failure, Loss of kidney function, and End-stage kidney disease criteria (or pRIFLE) as follows: estimated creatinine clearance — Schwartz formula, k × height (cm)/serum creatinine (mg/dL) (k = 0.33 in low birth weight < 1 year; 0.45 in full term < 1 year; 0.55 in 2-12 years of age and 13-18-year-old female; 0.7 in 13-18-year-old male) — value ≤ 75% of preoperative value or < 35 ml/min/1.73m^2^ and/or urine output < 0.3 ml/kg/hour for 24 hours or anuric for 12 hours^[17]^.

The surgical procedures were performed following a standard institutional protocol on the conduct of anesthesia and CPB. All patients were mechanically ventilated employing a pressure-controlled ventilation mode with a targeted arterial partial pressure of carbon dioxide within 35-45 mmHg. Methylprednisolone in a dose of 30 mg/kg intravenously was administered prior to the administration of CPB. The CPB circuit was primed with leukoreduced packed red blood cell, albumin, crystalloid (plasmalyte A), mannitol, sodium bicarbonate, and heparin. Heparin (4 mg/kg) was administered to achieve activated clotting time > 400 seconds. Following aortic and bicaval cannulation, CPB was instituted at a pump flow rate of 2-3 L/min/m^2^ of body surface area, maintaining hematocrit of 25-28%, moderate hypothermia (rectal temperature 28°-32° C), and perfusion pressure of 35-55 mmHg. Intravenous infusion of dopamine (5 µg/kg/min) and milrinone (0.5 µg/kg/min) were instituted to facilitate weaning from CPB. Ultrafiltration was performed during and after completion of CPB. Heparin was reversed with a slow infusion of protamine (in 1:1 ratio) over 10-15 minutes. The post-CPB blood and blood-product management were aimed at maintaining hematocrit > 30% and clinical judgement of the operative bleeding.

### Statistical Analysis

The categorical variables were expressed as number of patients and percentage of patients and compared between the groups using the Chi-square test. The continuous variables were expressed as mean and standard deviation and compared between the groups using the unpaired t-test. Herein, the Kolmogorov-Smirnov test was performed to assess normality. To measure the correlation between continuous variables, Pearson’s correlation analysis was performed. The non-parametric receiver operating characteristic (ROC) curve analysis was contemplated to evaluate the accuracy of various variables in predict poor outcomes indicated by their respective area under the curve (AUC). The “optimum cutoff point” was determined as the cutoff point with the highest ([sensitivity + specificity]/2) ratio, at which there was a maximal correct classification of the unfavorable outcomes. The sensitivity, specificity, and predictive values were reported using these generated cutoffs. The multivariate analysis was performed using binary logistic regression method. Analysis of variance test was used to do intergroup analysis. The statistical software IBM Corp. Released 2011, IBM SPSS Statistics for Windows, Version 20.0, Armonk, NY: IBM Corp. has been used for the analysis. An alpha level of 5% has been considered with any P-value < 0.05 considered as significant.

## RESULTS

Among the total of 1,040 patients included in the study, 180 patients (17.3%) developed poor outcome with an associated mortality rate of 2.8% (30 patients). The demographic data, perioperative characteristics, and preoperative laboratory parameters are listed in Table 1. The study patients were predominantly male with mean age of 24.91 months.

**Table 1:** Comparison of demographics, perioperative characteristics, and preoperative laboratory parameters between the favorable and poor outcome groups.

**Variable**		**Total patients**	**Favorable outcome**	**Poor outcome**	***P*-value**
**1. Demographic parameters**					
Age (months)		24.91±9.91	25.04±9.83	24.33±10.3	0.384
Sex	Female	413 (39.71%)	342 (39.77%)	71 (39.44%)	0.936
	Male	627 (60.29%)	518 (60.23%)	109 (60.56%)	
Weight (kg)		14.39±3.81	14.48±3.58	13.98±4.74	0.109
Height (cm)		75.59±6.26	75.7±5.95	75.05±7.56	0.202
Preoperative RVSP (mmHg)		52.34±16.2	49.01±14.41	68.25±14.78	< 0.001
Preoperative CCF		190 (18.27%)	118 (13.72%)	72 (40%)	< 0.001
Preoperative PH	Mild	594 (57.12%)	571 (66.4%)	23 (12.78%)	< 0.001
	Moderate	252 (24.23%)	219 (25.47%)	33 (18.33%)	
	Severe	194 (18.65%)	70 (8.14%)	124 (68.89%)	
**2. Perioperative characteristics**					
Diagnosis					
ASD		365 (35.1%)	304 (35.3%)	61 (33.8%)	0.699
VSD		590 (56.7%)	486 (56.5%)	104 (57.7%)	0.767
AVSD		85 (8.1%)	70 (8%)	15 (8.3%)	0.894
ACC time (mins)		52.62±9.02	49.93±6.28	65.46±9.09	< 0.001
CPB time (mins)		67.22±10.91	63.82±7.15	83.44±11.2	< 0.001
DO-MV (hours)		11.95±5.71	10.19±3.55	20.31±6.64	< 0.001
LOS-ICU (days)		2.4±1.78	1.85±1.15	5.03±1.88	< 0.001
LOS-H (days)		4.88±2.84	3.99±1.78	9.16±3.07	< 0.001
**3. Laboratory parameters**					
Hb (g/dL)		12.56±0.69	12.58±0.36	12.51±0.46	0.05
TLC (/mm3)		7070.71±725.55	7051.41±550.14	7126.77±754.06	0.119
NLR		3±0.69	2.81±0.52	3.9±0.68	< 0.001
PLR		113.31±33.9	104.47±24.68	155.53±39.75	< 0.001
SII		789.2±234.87	739.81±203.92	1025.15±230.11	< 0.001
Urea (mg/dL)		32.16±7.02	32.09±7.04	32.52±6.97	0.453
Creatinine (mg/dL)		0.8±0.15	0.8±0.14	0.79±0.17	0.605
Albumin (g/dL)		3.76±0.92	3.77±0.96	3.69±0.68	0.256

Data are presented as mean±standard deviation or number (%) ACC=aortic cross-clamping; ASD=atrial septal defect; AVSD=atrioventricular septal defect; CCF=congestive cardiac failure; CPB=cardiopulmonary bypass; DO-MV=duration of mechanical ventilation; Hb=hemoglobin; LOS-H=length of hospital stay; LOS-ICU=length of intensive care unit stay; NLR=neutrophil-lymphocyte ratio; PH=pulmonary hypertension; PLR=platelet-lymphocyte ratio; RVSP=right ventricular systolic pressure; SII=systemic immune-inflammatory index; TLC=total leukocyte count; VSD=ventricular septal defect

The poor outcome and the favorable outcome groups demonstrated statistically significant differences in preoperative clinical (RVSP [a measure of degree of PH] and CCF) and laboratory parameters (albumin, NLR, PLR, and SII) and intraoperative ACC and CPB times (Table 1). On univariate analysis, the aforementioned parameters predicted poor postoperative outcome (Table 2). However, subsequent to a multivariate analysis, preoperative RVSP (odds ratio [OR]: 0.690, 95% confidence interval (CI): 1.011-4.523, *P*<0.001), NLR (OR: 2.036, 95% CI: 1.36-3.037, *P*<0.001), SII (OR: 1.038, 95% CI: 1.016-1.059, *P*<0.001), ACC time (OR: 0.673, 95% CI: 0.523-0.866, *p*-0.002), and CPB time (OR: 1.937, 95% CI: 1.636-2.294, *P*<0.001) emerged as independent outcome predictors (Table 2).

**Table 2:** Univariate and multivariate logistic regression analyses of the preoperative risk factors for predicting postoperative poor outcome.

**Variables**		**Univariate analysis**				**Multivariate analysis**		
	**OR**	**95% CI**		***P*-value**	**OR**	**95% CI**		***P*-value**
		**Lower**	**Upper**			**Lower**	**Upper**	
Preoperative RVSP	24.990	16.765	37.249	< 0.001	0.690	1.011	4.523	< 0.001
Preoperative CCF	1.192	1.004	2.984	< 0.001	0.853	0.290	2.507	0.773
ACC time	1.234	1.201	1.267	< 0.001	0.673	0.523	0.866	0.002
CPB time	1.183	1.158	1.208	< 0.001	1.937	1.636	2.294	< 0.001
Albumin	0.904	0.760	1.076	0.256	2.036	1.365	3.037	0.543
NLR	4.192	2.937	5.984	< 0.001	2.036	1.365	3.037	< 0.001
PLR	1.058	1.049	1.068	< 0.001	1.021	0.602	1.732	0.938
SII	1.052	1.044	1.060	< 0.001	1.038	1.016	1.059	< 0.001

ACC=aortic cross-clamping; CCF=congestive cardiac failure; CI=confidence interval; CPB=cardiopulmonary bypass; NLR=neutrophil-lymphocyte ratio; OR=odds ratio; PLR=platelet-lymphocyte ratio; RVSP=right ventricular systolic pressure; SII=systemic immune-inflammatory index

**Fig. 2:**
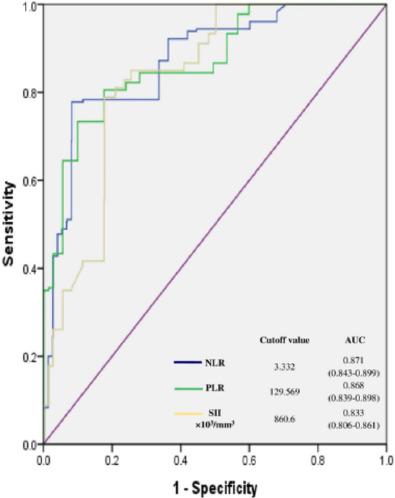
Illustration of the receiver operating characteristic curves for the postoperative poor outcome predictive value of the three hematological parameters (NLR, PLR, and SII) alongside the respective cutoff values and the area under the curve (AUC) depicted in the right lower panel. The respective confidence intervals (CI) for AUC are enlisted in parenthesis. NLR=neutrophil-lymphocyte ratio; PLR=platelet-lymphocyte ratio; SII=systemic immune-inflammation index

By ROC curve analysis, a cutoff of 3.332 for NLR, 129.569 for PLR, and 860.605×10^3^/mm^3^ for SII significantly predicted poor outcome with sensitivity, specificity, and AUC as follows: 77.8%, 91.7%, 0.871; 73.3%, 90%, 0.868; and 78.9%, 82.2%, 0.833, respectively (*P*<0.001) (Figure 2).

On secondary analysis, the postoperative variables like mean VIS, DO-MV, LOS-ICU, and LOS-H were significantly higher in the poor outcome group (*P*<0.001) as enlisted in Table 1. On Pearson’s correlation study, all three hematological indices (NLR, PLR, and SII) significantly correlated with mean VIS, DO-MV, LOS-ICU, and LOS-H wherein the correlation coefficient R was higher for NLR (R = 0.697 for mean VIS, 0.91 for DO-MV, 0.89 for LOS-ICU, and 0.91 for LOS-H), as depicted in Table 3.

**Table 3:** Correlation study of NLR, PLR, and SII with mean VIS, DO-MV, LOS-ICU, and LOS-H.

	**NLR**		**PLR**		**SII**	
	**R value**	***P*-value**	**R value**	***P*-value**	**R value**	***P*-value**
Mean VIS	0.697	< 0.001	0.516	< 0.001	0.484	< 0.001
DO-MV	0.917	< 0.001	0.651	< 0.001	0.662	< 0.001
LOS-ICU	0.895	< 0.001	0.633	< 0.001	0.647	< 0.001
LOS-H	0.914	< 0.001	0.654	< 0.001	0.669	< 0.001

R value = Pearson’s correlation coefficient DO-MV=duration of mechanical ventilation; LOS-H=length of hospital stay; LOS-ICU=length of intensive care unit stay; NLR=neutrophil-lymphocyte ratio; PLR=platelet-lymphocyte ratio; SII=systemic immune-inflammatory index; VIS=vasoactive-inotropic score

The intergroup analysis elucidated that the NLR, PLR, and SII values were significantly higher in patients with preoperative severe PH compared to those having mild and moderate degree of PH (stratified in accordance to the preoperatively documented RVSP) (*P*<0.001) (Table 4). Moreover, NLR, PLR, and SII values were also found to be significantly higher in subjects with poor outcome as compared to those with favorable outcome, in all the three subgroups classifying as mild, moderate, and severe PH (Table 4).

**Table 4:** Comparison of preoperative NLR, PLR, and SII amongst the mild, moderate, and severe PH subgroups and intra-subgroup NLR, PLR, and SII comparison as per the involved postoperative outcome.

		**Mild PH**	**Moderate PH**	**Severe PH**	***P*-value**
**NLR**	**Overall**	2.54±0.11	3.08±0.16	4.32±0.15	*< 0.001*
	**P, F**	2.63±0.11, 2.53±0.11	3.16±0.26, 3.06±0.14	4.43±0.16, 4.32±0.14	
	**(*P*-value)**	*(< 0.001)*	*(< 0.001)*	*(0.001)*	
**PLR**	**Overall**	95.90±21.14	114.86±12.45	164.60±32.11	*< 0.001*
	**P, F**	114.11±8.38, 95.16±21.18	120.23±13.49, 114.66±12.31	173.67±34.24, 148.53±19.63	
	**(*P*-value)**	*(< 0.001)*	*(0.01)*	*(<0.001)*	
**SII (×10^3^/mm^3^)**	**Overall**	648.05±132.15	844.25±810.18	1149.88±196.86	*< 0.001*
	**P, F**	803.28±372.97, 641.79±130.77	879.44±692.72, 844.97±827.59	1210.37±220.99, 1115.73±124.64	
	**(*P*-value)**	*(< 0.001)*	*(0.025)*	*(0.001)*	

Data are presented as mean±standard deviation F=favorable outcome; NLR=neutrophil-lymphocyte ratio; P=poor outcome; PH=pulmonary hypertension; PLR=platelet-lymphocyte ratio; SII=systemic immune-inflammatory index

## DISCUSSION

The index study outlined preoperative NLR, PLR, and SII as significant prognostic hematological indices for predicting poor outcome following corrective cardiac surgery in ACHD patients with pre-existing PH along with other clinical and demographic parameters (preoperative RVSP, ACC, CPB time) in Cox’s univariate analysis. Nevertheless, NLR and SII remained as independent hematological predictors of poor outcome on multivariate analysis. In addition, preoperative NLR and SII values positively correlated with mean VIS, DO-MV, LOS-ICU, and LOS-H. Interestingly, the platelet-leukocyte indices were significantly increased in patients with poor outcomes compared to those with favorable outcomes, in the context of subgroup classification of ACHD-PH patients into mild, moderate, and severe PH premised on the extent of preoperative RVSP elevation.

The absence of significant difference in the preoperative TLC between the favorable and poor outcome patients supports the hypothesis of the index study proposing an improved prognostic potential of differential leukocyte indices. This is in accordance with the elucidation of the superiority of NLR over TLC in previous outcome predictive studies by Gurm et al.^[18]^ and Madjid et al.^[19]^ in coronary artery disease patients (non-operative settings) and by Gibson et al.^[9]^ in his pioneer study involving patients undergoing surgical revascularization. The study by Gibson et al.^[9]^ also deduced a NLR cutoff value of 3.36 for predicting mortality following surgical revascularization. Another research by Savluk et al.^[13]^ demonstrated a mean NLR value of 2.84 to be associated with increased mortality following hypoplastic left heart surgery. These cutoff values are in close proximity to the derived NLR cutoff value of 3.33 in the present study. Moreover, Manuel et al.^[14]^ highlighted the association between higher preoperative NLR value and increased DO-MV, LOS-ICU, and LOS-H in pediatric patients undergoing bi-directional Glenn shunt, which is in harmony with the findings of the index study outlining a significant correlation of leukocyte indices with the aforementioned postoperative parameters, in addition to VIS.

PLR, which constitutes another novel hematological parameter (a platelet-leukocyte index serving as a combined inflammatory--aggregatory marker), has been evaluated for outcome predictive value in adult cardiac surgical settings. However, there is dearth of literature on the prognostic role of PLR in congenital cardiac surgical cohort. Nevertheless, two retrospective studies in adult patients undergoing surgical revascularization have demonstrated the association of increased PLR with individual postoperative complications — Gungor et al.^[20]^, highlighting an accentuated risk of postoperative atrial fibrillation with a preoperative PLR > 119.3, and Parlar and Saskin^[21]^, revealing the association between a mean PLR of 154.5 with predisposition to postoperative AKI —. The present study also derived a PLR cutoff value of 129.569 as a predictor of poor composite outcome in contrast to the existing literature focusing on independent evaluation of specific postoperative complications.

Recent endeavors aimed at evaluating the prognostic value of another novel hematological index, SII, originally described in oncological settings^[22]^, have also yielded promising results in cardiac patients^[10]^. Regarding the existing SII cutoffs in cardiac patients, Yang et al.^[23]^ depicted an increased incidence of post-percutaneous coronary intervention major cardiovascular events with a SII value > 694.3×10^9^. On the other hand, Agus et al.^[12]^ attributed a mortality predictive potential to the SII value > 2314 in infective endocarditis patients. However, there is a relative scarcity of evidence evaluating the role of SII in operative cardiac setting, with the seminal study of Dey et al.^[10]^ highlighting poor postoperative outcomes in off-pump coronary artery revascularization cohort with a preoperative SII ≥ 878.06×10^3^/mm^3^. Our evaluation of the prognostic role of SII in congenital cardiac surgical computed a predictive SII cutoff value of 860.605×10^3^/mm^3^ signifying the role of inflammation in the underlying pathophysiology of PH associated with ACHD. Albeit comparable to Dey et al.^[10]^ and Yang et al.^[23]^, the index SII cutoff was considerably lower in comparison to the Agus et al.^[12]^ cutoff given the proven infective etiology in most of the patients in their study.

Moreover, the inclusion of cardiac surgical patients with preoperative PH in the index study was noteworthy, particularly motivated by the accumulating literature on the prognostic role of the novel hematological indices in diverse PH settings. In this context, a unique study by Zuo et al.^[15]^ discovered significantly elevated NLR, PLR, and SII values in patients complicated with PH in acute exacerbation of COPD. The aforementioned study also investigated the values of three indices among the mild, moderate, and severe PH groups of the study population. Although NLR and SII values were elevated in the severe PH group, only an increased PLR value in the severe and moderate groups as compared to the mild PH group reached the level of significance (*P*-value=0.01). However, the present study demonstrated significantly higher values of all the three study indices (NLR, PLR, and SII) in the severe and moderate PH groups as compared to the mild PH groups.

The importance of a combined assessment of the corpuscular lines in an inflammatory process outlines the role of composite platelet-leukocyte indices as depicted in the index study. Talking of the leukocytes, while the association of neutrophilia with ongoing inflammation is well known, an improved recent understanding of the stress-related relative lymphocytopenia (owing to a catecholamine-cortisol surge, apoptotic cell death, peripheral redistribution, and down-regulation of CD4^+^ T-cells) further substantiates the role of these novel indices^[24]^. Alongside inflammation in PH, the endothelial dysfunction associated with PH may also potentially result in an enhanced procoagulant activity, inappropriate fibrinolysis, and platelet activation^[1]^. Herein, the evolving reputation of platelets as important mediators of the cross talk between the immune system, endothelial cells, and the coagulation pathways adds to the contextual significance in settings like PH-predisposed endothelium functional disturbances. Moreover, the recent proposition of envisaging PH as a complex interaction between the inflammatory, coagulation, and complement pathways provides additional physiological premise for the outcome predictive study observations.

### Strength and Limitations

Firstly, the study constitutes a novel endeavor of investigating the prognostic role of cost-effective and readily available hematological indices like NLR, PLR, and SII as inflammatory markers in ACHD patients with PH. Secondly, a large sample size bestows additional merit to the study. Lastly, the evaluation of a composite outcome (with objective definitions of postoperative complications) is classified as a study strength. The study had a few limitations. First and foremost, the retrospective nature of the study renders the observations susceptible to a range of confounding factors. Secondly, the present study was conducted in a single tertiary cardiac center wherein the institutional management protocol can also have potential confounding impact. Thirdly, a 30-day follow-up period following the surgery limited the evaluation to the involved short-term outcomes. Lastly, we did not assess the prognostic value of the postoperative platelet-leukocyte indices (retrospectively outlined in a congenital cardiac setting by Xu et al.^[25]^) and the collaborative value in conjunction with the known markers of inflammation such as C-reactive protein (CRP) (not available in our patient records) particularly when a few independent researchers have delineated a correlation between CRP and NLR^[26]^.

## CONCLUSION

Novel parsimonious and reproducible hematological indices present the potential of assisting the risk stratification of congenital cardiac surgical patients with pre-existing PH. Given the recognition of PH being an intriguing pathobiophysiological process staging the complex interactions amongst the corpuscular lines, we infer that these prognostic hematological indices can facilitate the subsequent modulation of the anesthetic-perfusion-surgical conduct to minimize the inflammatory sequel in order to ensure favorable postoperative outcomes.
